# Late Diagnosis of Complete Colonic and Rectal Duplication in a Girl with an Anorectal Malformation

**DOI:** 10.1055/s-0039-1692193

**Published:** 2019-07-05

**Authors:** Amr AbdelHamid AbouZeid, Shaimaa Abdelsattar Mohammad, Sherif Elhussiny Ibrahim, Anas Fagelnor, Ahmad Zaki

**Affiliations:** 1 Department of Pediatric Surgery, Ain Shams University, Abbasia, Cairo, Egypt; 2 Department of Radiodiagnosis, Ain Shams University, Cairo, Egypt; 3 Department of Pediatric Surgery, Benha Children Hospital, Benha, Egypt; 4 Department of Pediatric Surgery, Ain Shams University, Abbasia, Cairo, Egypt

**Keywords:** rectoperineal fistula, cloaca, anorectal malformation, MRI

## Abstract

Complete colonic duplication is rare, and usually occurs as a part of the caudal duplication syndrome. In such cases, the diagnosis is clinically evident by the presence of two ani arranged side by side in the perineum, which is commonly associated with duplication of the external genitalia as well (double phallus or double vestibule). In this report, we present a special case of anorectal anomaly that was associated with complete tubular colonic duplication. The diagnosis was initially missed due to the uncommon sagittal arrangement of duplicated rectum: one rectum was ending externally into the perineum by rectoperineal fistula, while the other was hidden by its internal termination into the vagina. Our final diagnosis for this case was a variant of anorectal anomaly in the female, which was associated with complete colonic duplication. One colon (which was in the free mesenteric border) terminated anteriorly into the vagina as a part of a “short common channel” cloaca, while the other colon terminated by rectoperineal fistula. Although the anomaly seems to be rather complex and confusing, yet our case was associated with an excellent outcome due to the benign type of anorectal anomalies (rectoperineal fistula and “short common channel” cloaca) and absence of significant sacral dysplasia; in addition to adequate identification of the abnormal anatomy by appropriate investigations and the staged approach for surgical reconstruction.

## Introduction

Anorectal Anomalies (ARA) represent a major category in pediatric surgery with different phenotypes in the male and female. 1 Successive classifications have been proposed trying to include the wide diversity of the spectrum; and lastly, the Krickenbeck conference identified ARA as seven major clinical groups and other rare variants. 2

Here, we present a special case of ARA in which the clinical picture was further complicated by the presence of complete tubular colonic duplication.

## Case Presentation

A 2-year-old girl presented by abnormal passage of stools through her vestibule. She had a history of operation (anorectoplasty) during the neonatal period. The patient operative files revealed a limited sagittal anorectoplasty performed (without colostomy) for a rectoperineal fistula, with uneventful postoperative recovery.

The history would suggest an iatrogenic rectovaginal fistula, and, therefore, the patient was scheduled for examination under anesthesia with the possibility of performing a diverting colostomy. Examination under anesthesia revealed a well-positioned neoanus with good caliber; however, the vestibule showed a shallow common urogenital sinus with a septated vagina. The common sinus was short and wide that the anatomy was well-exposed for inspection upon labial retraction. Stools were seen emerging out of the vagina upon applying pressure on the lower abdomen. A decision was taken for fecal diversion as a first step for a staged repair. An oblique left lower abdominal incision was made to perform a pelvic (sigmoid) colostomy which revealed the presence of double colon sharing a common mesentery. A pelvic colostomy was done for the double colon that consisted of two proximal stomas and two distal mucous fistulae.

**Fig. 1:**
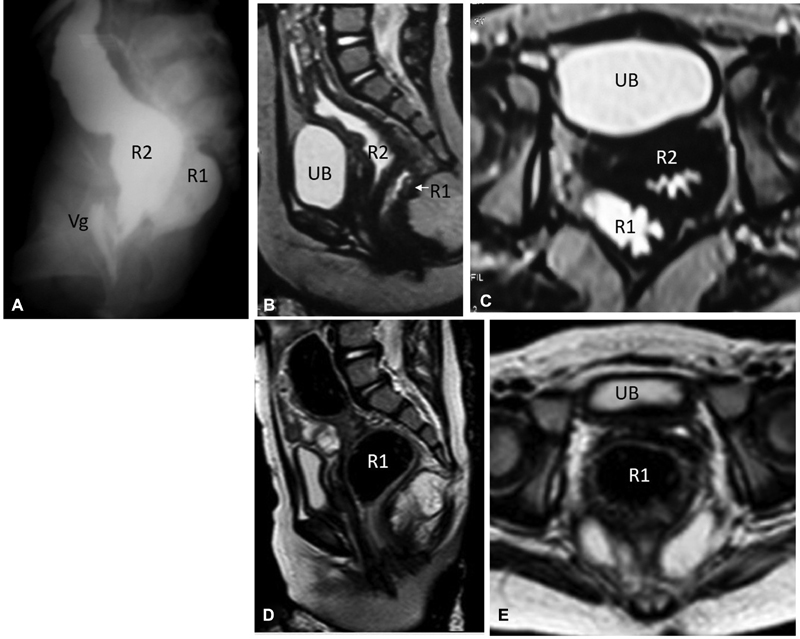
A 2-year-old girl with complete colonic duplication on top of anorectal anomaly. The upper row demonstrates investigations performed before reconstruction: ( **A** ) contrast studies through the two distal “colostomy” stomas demonstrating one posterior colon and rectum (R1) terminating by the neo-anus, and another anterior colon and rectum (R2) terminating into the vagina (Vg); ( **B** ) and ( **C** ) midsagittal and axial MRI (T2-WI), respectively, demonstrating the orientation of both recti (R1 and R2) in relation to each other and to other pelvic organs (UB, urinary bladder). Note the injection of gel through both recti to facilitate their identification in MRI by the hyperintense signal of the gel in T2-WI. The lower row demonstrates pelvic MRI anatomy following reconstruction (after excision of anterior rectum R2, and closure of colostomy); ( **D** ) midsagittal plane; ( **E** ) axial plane. MRI, magnetic resonance imaging; WI, weighted image.

*Diagnostic workup before definitive repair:* we performed contrast X-ray studies ( Fig. 1A ) and pelvic magnetic resonance imaging (MRI; Fig. 1B and C ). Injection of contrast through the proximal stomas (each at a time) revealed complete colonic duplication up to a double cecum (with failure of contrast to pass into neither the ileum nor the appendix). Injection of contrast through the distal mucous fistulae revealed one posterior colon and rectum (R1) terminating by the neoanus, and another anterior colon and rectum (R2) terminating via a rectovaginal fistula ( Fig. 1A ). MRI studies revealed no associated spinal anomalies; and helped to complete the picture by defining the three-dimensional (3D) orientation of the double colon and rectum in relation to other pelvic soft tissue structures ( Fig. 1B and C ).

The sacral ratio was calculated in the antero–posterior X-ray film of the sacrum; its value was 0.53, which indicated a fair prognosis regarding the continence potential. 3

**Fig. 2:**
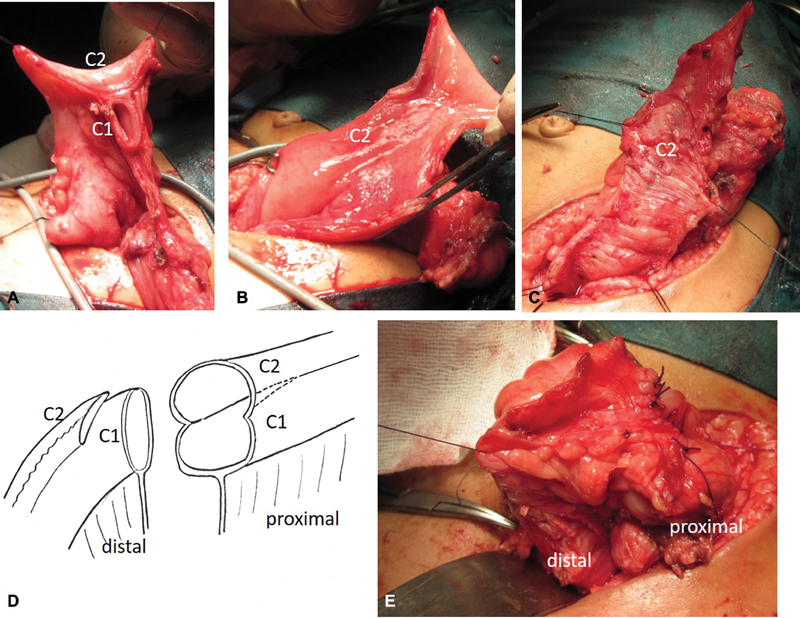
Intraoperative photos and diagram representing extra-mucosal excision of the antimesenteric distal colon (C2) and rectum. ( **A** ) The distal colon to be excised (C2) was incised along its free antimesenteric border; ( **B** ) submucosal injection of adrenaline/saline solution (1/200,000) to control bleeding and elevate the mucosa (hydrodissection); ( **C** ) stripping of the mucosa off this distal colon and rectum; ( **D, E** ) the continuity of the fecal stream was then restored by anastomosing the proximal double colonic lumens with the retained distal colon (C1) in a Y-shape fashion. Note the limited terminal septotmy for the common wall between the proximal double colonic lumens in ( **D** ).

*Operative management:* our surgical plan was to remove the distal colon and rectum (R2) communicating with the vagina, while keeping the posterior distal colon and rectum (R1) as a single exit for fecal stream after reanastomoses with proximal double colon ( Fig. 2D ).

As expected, separation of one colon from the other was unfeasible due to their common mesentery, so we went for just mucosal excision of the “antimesenteric” distal colon and rectum (R2). The distal colon to be excised was incised along its free antimesenteric border ( Fig. 2A ), followed by submucosal injection of adrenaline/saline solution (1/200,000) to control bleeding and elevate the mucosa (hydrodissection; Fig. 2B ). Stripping the mucosa off this distal colon and rectum was then applied down into the pelvis; the stripped mucosa was transfixed and excised at its lower end communicating into the vagina ( Fig. 2C ). This was followed by seromuscular closure. The continuity of the fecal stream was then restored by anastomosing the proximal double colonic lumens with the retained distal colon and rectum in a **Y** -shape fashion ( Fig. 2D and E ). Before that, a 7 cm linear cutting stapler was applied to the distal end of the common wall between the proximal double colonic lumens (limited septotomy), converting it into a single wide lumen that could be anastomosed to the single distal colon ( Fig. 2D ). Postoperative recovery was uneventful, and the patient had normal defecation through a single neoanus. Few months later, the patient underwent partial urogenital sinus mobilization with division of the vaginal septum as a final step in the staged reconstruction. Partial urogenital sinus mobilization (PUM) is a procedure used to correct short common urogenital sinus anomalies which comprises dissection of the common urogenital sinus up to but without cutting through the pubourethral ligament. 4

The patient has been followed-up for the last 3 years, with a satisfactory functional and cosmetic outcome. She has voluntary fecal and urinary control; occasional constipation showed good response to stimulant laxatives. A follow-up MRI has been prescribed to check for postreconstruction pelvic anatomy ( Fig. 1D and E ).

## Discussion

Complete colonic duplication is rare and usually occurs as a part of the caudal duplication syndrome. 5 In such cases, the diagnosis is clinically evident by the presence of two ani arranged side by side in the perineum which is commonly associated with duplication of the external genitalia as well (double phallus or double vestibule). 6 In this report, the diagnosis was initially missed due to the uncommon sagittal arrangement of duplicated rectum: one rectum was ending externally into the perineum by apparent rectoperineal fistula, while the other was hidden by its internal termination into the vagina. The patient was initially managed as a straight forward case of rectoperineal fistula by primary limited sagittal anorectoplasty. Later, the other hidden colon showed itself by passage of stools through the vagina, when we suspected an iatrogenic rectovaginal fistula complicating the primary operation. A decision for fecal diversion was taken, and, surprisingly, we detected the presence of double colon at time of colostomy creation. Conventional contrast studies confirmed the diagnosis; MRI provided anatomical soft tissue details and could have been useful for making the correct diagnosis prior to abdominal exploration.

Another point that was missed during the initial management of this case was the presence of associated urogenital anomaly in the form of a short common urogenital sinus and a septated vagina. This highlights the importance of proper exposure (labial retraction) during inspection that would help to identify all aspects of the anomaly. A short common channel in cloaca (< 1 cm) has been reported as a benign type with excellent prognosis. 7 This can be managed either by simple introitoplasty without dissection around the urethra 7 or by partial urogenital sinus mobilization (PUM). 4 On the other hand, division of vaginal septum has been recommended to be performed early during anorectal reconstruction to avoid possible psychological concerns of such procedure when performed during adolescence. 8

Our final diagnosis for this case was a variant of anorectal anomaly in the female, which was associated with complete colonic duplication. One colon (which was in the free mesenteric border) terminated anteriorly into the vagina as a part of a ‘short common channel’ cloaca (with rectovaginal communication); 9 while the other colon terminated via a rectoperineal fistula. Although the anomaly seems to be rather complex and confusing, yet our case was associated with an excellent outcome due to the benign type of anorectal anomalies (rectoperineal fistula and “short common channel” cloaca) 7 and absence of significant sacral dysplasia; 3 in addition to adequate identification of the abnormal anatomy by appropriate investigations and the staged approach for surgical reconstruction.

## Notes

New Insights and the Importance for the Pediatric Surgeon The present article describes how to manage a rare case of anorectal anomaly associated with complete colonic duplication.
